# All-Dielectric Multilayer Cylindrical Structures for Invisibility Cloaking

**DOI:** 10.1038/srep09574

**Published:** 2015-04-10

**Authors:** Ali Mirzaei, Andrey E. Miroshnichenko, Ilya V. Shadrivov, Yuri S. Kivshar

**Affiliations:** 1 Nonlinear Physics Centre, Research School of Physics and Engineering, The Australian National University, 59 Mills Rd, Acton, ACT, 2601, Australia

## Abstract

We study optical response of all-dielectric multilayer structures and demonstrate that the total scattering of such structures can be suppressed leading to optimal invisibility cloaking. We use experimental material data and a genetic algorithm to reduce the total scattering by adjusting the material and thickness of various layers for several types of dielectric cores at telecommunication wavelengths. Our approach demonstrates 80-fold suppression of the total scattering cross-section by employing just a few dielectric layers.

The possibility to suppress the total scattering cross-section of an object has attracted significant attention in recent years. One of the approaches to achieve nearly ideal invisibility is based on the so-called Transformation Optics^1^,^2^,^3^, which relies on a coordinate transformation. Using transformation optics necessitates the use of materials having inhomogeneous anisotropic permittivity and permeability. This is not achievable without using metamaterials^4^,^5^, which normally have narrow bandwidth, high losses and other practical complications for optical frequencies. Therefore other approaches were developed including using non-magnetic materials^6^, carpet cloaks^7^, optical ‘Janus’ devices^8^ and flattened Luneburg lenses^9^.

Another technique is scattering cancellation with plasmonic or dielectric multilayer structures. One of the first examples was demonstrated by Kerker for core-shell spherical particles consisting of metallic core and dielectric shell^10^. The out-of-phase electric dipole polarisabilities of subwavelength metallic and dielectric parts lead to total scattering cancellation in the far-field. Later this approach was widely used for various layered plasmonic structures^11^,^12^,^13^,^14^,^15^,^16^,^17^. It has been shown that by using this approach and employing normal materials (isotropic, homogeneous and non-magnetic media), the scattering cross section (SCS) can be minimized, thus providing invisibility of perfect electric conductor (PEC) cores. This is mostly achieved by a considerable number of layers^18^,^19^,^20^,^21^. Cloaking by a few layers of lossless, epsilon-near-zero materials^22^ was also reported to cloak a PEC core with a plasmonic structure^21^ which is not yet experimentally available. Generally, plasmonic materials make it easier to control the SCS, but they introduce prohibitively high losses near plasmonic resonance frequencies. This issue becomes less crucial far from the resonances, where reduction of the SCS can also be achieved^23^. One of the solutions to this problem is the use of nearly lossless dielectric materials to control SCS, and this is the focus of this paper.

The possibility to realize a structure which is invisible in the optical range raises several important questions. (i) Is it possible to use real conventional materials to control SCS practically? (ii) How many layers are required to achieve the best invisibility possible? (iii) Can we design all-dielectric structures to exhibit lossless invisibility in the optical range? In this work we answer these questions by employing a genetic algorithm to optimize SCS properties of a dielectric cylinder. We use experimental data for some commonly used dielectrics as well as for the materials used in the shells, and show that one can achieve up to 80 fold total scattering cross-section suppression. We find that the optimum invisibility is achievable with three or even smaller number of dielectric shells.

## Results

**Figure 1:**
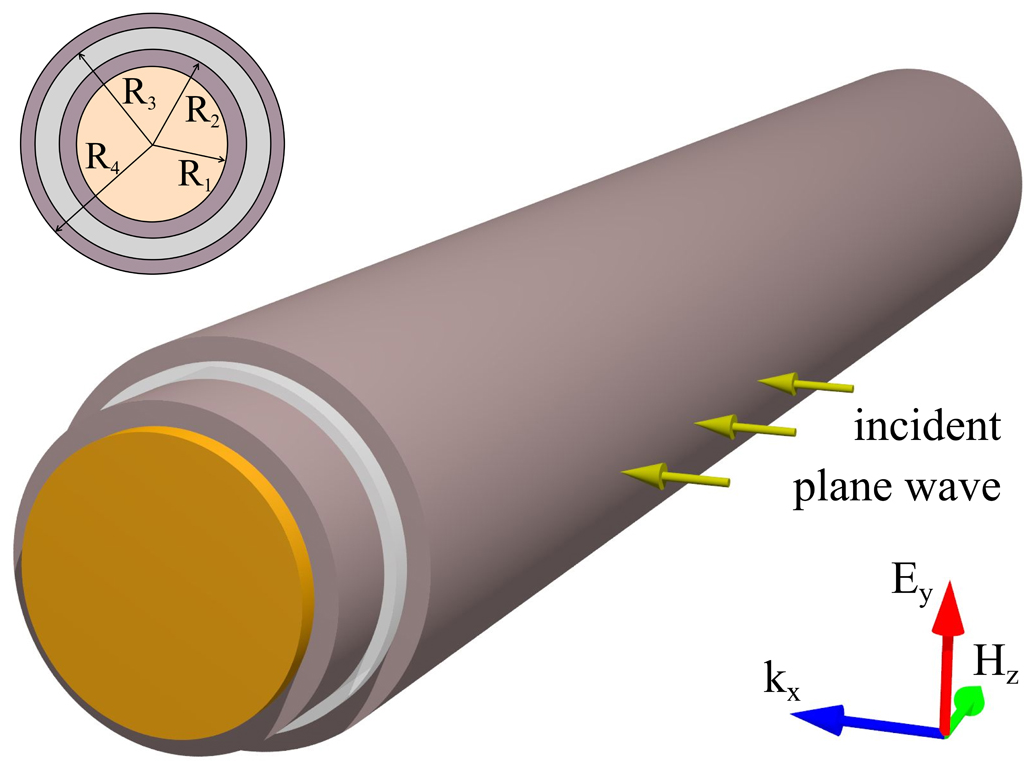
Geometry of the problem: cloaking of a cylindrical scatterer with a multi-layer all-dielectric coating. The plane wave is incident from the side and the magnetic field is parallel to the structure's axis.

Figure 1 shows general schematics of the problem, a cylindrical multilayer structure illuminated by a plane wave incident normal to the structure axis. We use telecommunication wavelength of 1550 nm and consider three different materials, AlAs, TlBr and GaAs^24^,^25^ for the core.

The incident electromagnetic plane wave is propagating along the *x* axis with the magnetic field polarized along the cylinder axis (similar results are presented for the orthogonal polarization in ‘Additional information’ section), 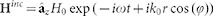, where **â**_z_ is a unit vector along z axis, and *H*_0_ is the amplitude of the incident magnetic field. We assume that our multi-layer structure contains *L* layers, and it is placed in free space. By decomposing the electromagnetic fields into a superposition of cylindrical harmonics, the field in layer *l* is presented as^26^ 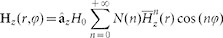, 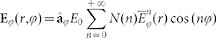 and 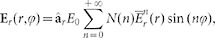 in which 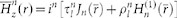, 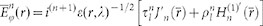, and 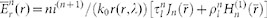, where 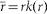, 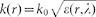, *J_n_* and 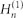 are the *n*-th order Bessel and Hankel functions of the first kind, respectively; *n* is the mode number, *r* is the radial coordinate within the *l*-th layer, 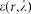 is the dielectric constant at each point in space for a given wavelength, 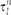 and 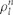 are the *n*-th mode coefficients in the *l*-th layer which will be found by solving the boundary condition equations for the tangential components of the fields, 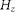 and *E_ϕ_*^27^. Additionally, we set 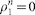 to avoid the singularity of the Hankel functions at the cylinder axis, and 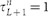 for each *n*, so that our equation also describes the incident plane wave as a superposition of cylindrical waves. Coefficient *N*(*n*) = 1 for *n* = 0 and for all other modes *N*(*n*) = 2 due to the symmetric relation of the Bessel functions with positive and negative indices.

By taking into account 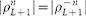 degeneracy, the SCS of the structure can be expressed as a function of mode amplitudes as^27^,^28^
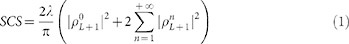
We then normalize all SCS values to 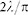, which we denote as the normalized SCS (NSCS).

**Figure 2:**
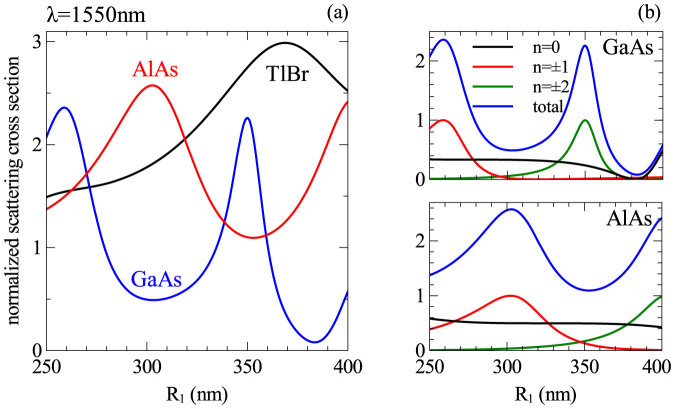
(a) Dependence of the normalized scattering cross-section on the cylinder radius for three different materials at 1550 nm, and (b) decomposition of the total scattering cross-section into contributions from different multipoles for GaAs and AlAs nanowires. The radii of nanowires are chosen such that their NSCS reaches maximum at 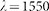 nm (*R_AlAs_* = 303 nm, *R_TlBr_* = 369 nm and *R_GaAs_* = 261 nm).

Invisibility implies that the SCS of the whole structure is minimized. We aim to achieve this by the proper design of multilayer dielectric shells. Here we use a genetic optimization algorithm^29^,^30^,^31^,^32^ (GA) to optimize the optical properties. To do so, we employ our GA to search for the optimal values of materials permittivity as well as layers' radii to minimize the total scattering at a given wavelength. We first define the material and the radius of the scatterer (core) as the input of our optimization process. A set of materials is also defined according to the working wavelength (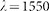 nm) and experimental data^24^,^25^ from which the GA chooses dielectric constants randomly to form the layers in the shell. We aim to suppress the total SCS of the resonant dielectric core. We choose a radius of the core *R*_1_ such that the nanowire exhibits the maximum of SCS at 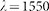 nm. Figure 2(a) shows the normalized scattering cross section as a function of nanowires' radius for different materials. Figure 2(b) shows the decomposition of the SCS into contributions from different order modes, namely monopole, dipole and quadrupole. Contributions from higher order modes are negligible.

**Figure 3:**
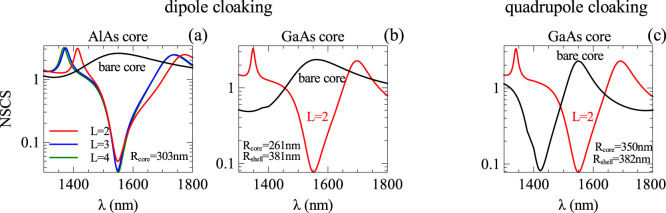
Suppression of the total scattering cross section for two different core materials. The core radii are chosen based on Fig. 2 to have the maximum SCS to be cancelled. (a,b) Demonstration of SCS suppression for dipolar resonance and (c) similar results for quadrupole cloaking, indicating the efficiency of the approach for different multipolar orders.

The genetic algorithm uses a database of several dielectric materials, and it chooses the ones that are more suitable for cloaking. We noticed that the GA always chooses two types of materials with the lowest and highest dielectric constants to form the highest possible index contrast between shell layers, which in our database are fused-silica and silicon. Materials with dielectric constant values in between are not chosen by the GA and adding such materials to our set does not help improving the cloaking quality. Figure 3 shows the reduction of the SCS that can be achieved by coating the nanowires by dielectric layers. Black curves correspond to the SCS of the nanowire without shells. Figure 3(a) shows how the scattering cross-section of AlAs nanowire can be reduced by adding more shells. Even 1 shell (L = 2) reduces the SCS by a factor of 50. Adding up to 3 shells allows the SCS to be reduced by 80 times. Figure 3(b) demonstrate that the SCS of the GaAs nanowire can be reduced by a factor of 30. In both cases the main contribution to the scattering cross-section is given by the dipole mode excitation in the structure. To show the wide applicability of the GA approach, we also consider a thicker GaAs core with radius of 350 nm. For such a nanowire, at the telecommunication wavelength, the main contribution to the SCS is given by the quadrupole mode. Figure 3(c) shows that the scattering induced by the quadrupole mode can be also suppressed by an appropriate choice of cloaking shell. The detailed optimization results for dipolar cloaking are summarized in Table 1. We note that for a simple core-shell structure, analytical expressions exist for the optimum shell size to compensate the SCS induced by individual modes, however such expressions do not exist for the total SCS, nor for the case of multilayer structures. Therefore our approach provides a more universal solution to cloaking optimization problems.

## Discussion

**Figure 4:**
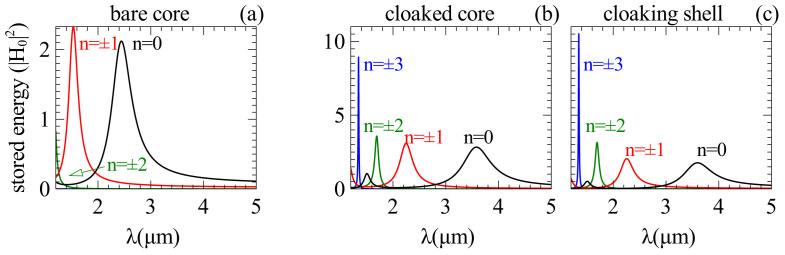
Stored energy. The values are normalized to |*H*_0_|^2^ and shown inside (a) the bare core, (b) the cloaked core and (c) the cloaking shell. Coating the silicon layer causes a red-shift in the resonance of all modes.

**Figure 5:**
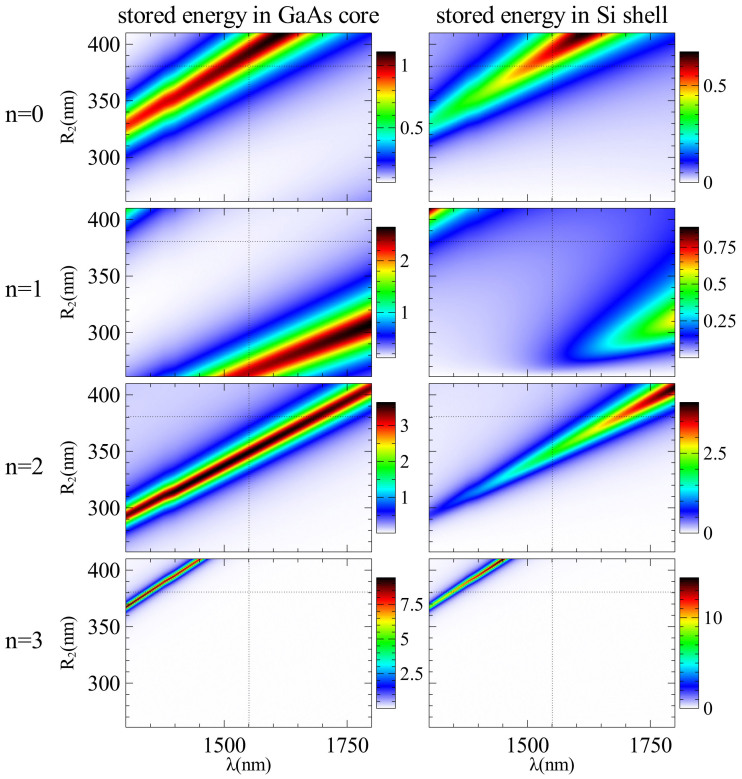
Spectral variation of stored energy of different modes in GaAs-Si core-shell structure with changing *R*_2_. In the plots, *R*_2_ starts from 261 nm, which indicates zero thickness of the shell (bare core).

**Figure 6:**
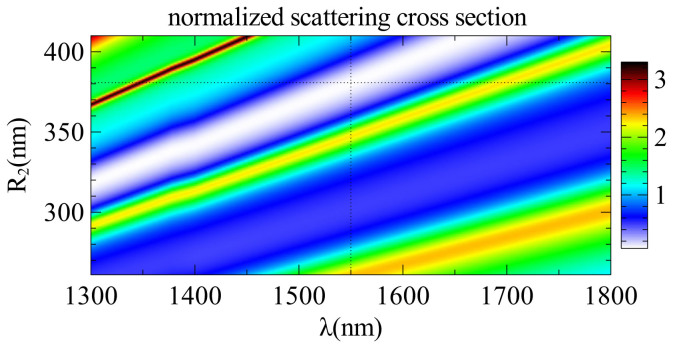
Spectrum of the scattering cross-section with changing *R*_2_. The shell radius starts from 261 nm which corresponds to the bare core structure. Dotted lines show the optimal parameter values.

Now we would like to understand the physical origin of the observed invisibility. One of the obvious reasons could be that the reduction of scattering is caused by a shift of the resonance of the core caused by adding a dielectric layer. To have more in-depth physical interpretation of the optimization process, we calculate the stored energy inside the core and the shell of the introduced GaAs-Si core-shell structure separately for different modes, as demonstrated in Fig. 4. Indeed, if we take a single dielectric shell and change its radius continuously, as presented in Fig. 5, all the resonances linearly shift with the shell thickness. Importantly, the optimal minimal SCS [see Fig. 6] is achieved near the resonant conditions of both core and shell as indicated by the dotted lines in Figs. 5 and 6. The peculiarity is that while the initial resonances are shifted towards longer wavelengths, different resonances appear at the given wavelength [see Fig. 4]. Similar to Kerker's original approach^10^, we have two out-of-phase resonant modes excitations of the core and shell which compensate each other in the far-field, resulting in scattering cancellation, with one important difference that now we are dealing with dielectric materials only.

Table 1 also illustrates that by adding more layers, the SCS saturates. Values shown in red in the table indicate configurations found by the GA that repeat results obtained for a smaller number of coating layers. After obtaining the optimized design (the green numbers in the table), our GA merges newly added layers to the previously existing ones and decreases the number of layers to the optimized design. In case of the GaAs scatterer, this saturation happens by coating the first silicon layer. The level of invisibility of the AlAs and TlBr cores saturates when adding three dielectric layers. This is in contradiction with the effective medium theory (EMT) for cloaking with anisotropic materials which predicts better invisibility with increasing number of isotropic layers^33^,^34^,^35^.

**Figure 7:**
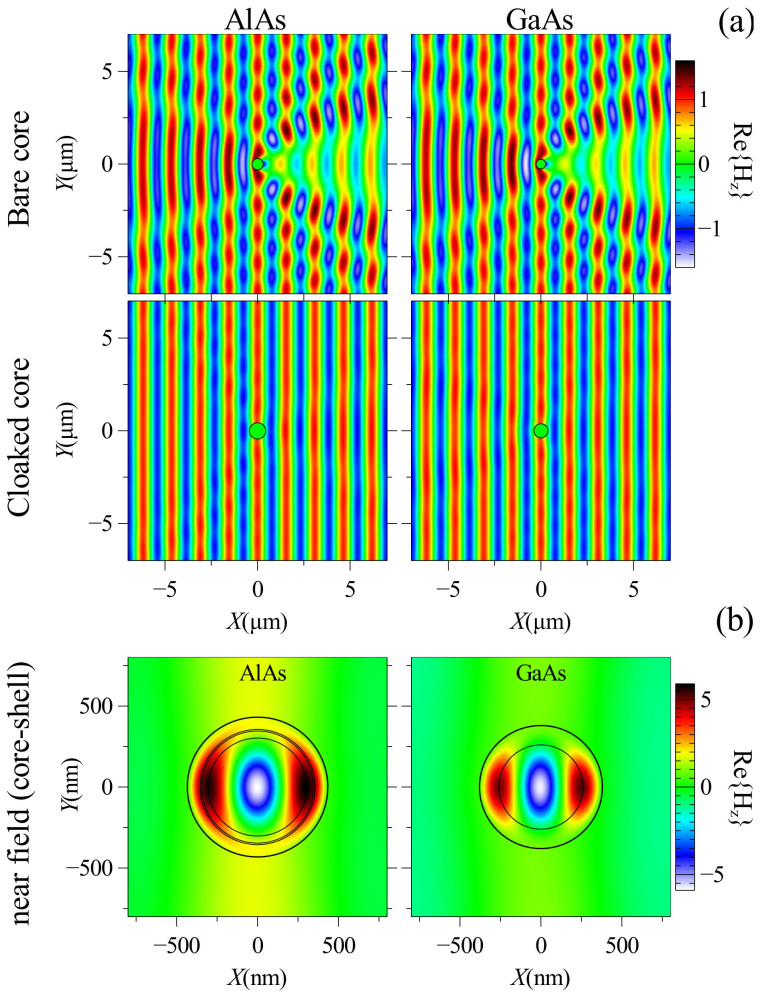
(a) Far field profiles of magnetic field for bare and invisible nanowires and (b) the field profiles inside the structures in invisibility regime. Optimized radii of core-shell structures are illustrated in Table 1.

The field profiles of bare nanowires and core-shell structures with cores made of AlAs and GaAs are calculated in both near and far field regions using the optimized parameters from Table 1 and they are shown in Fig. 7. The comparison between the far field profiles in bare and invisible structures reveals the drastic suppression of scattering and shows how the invisibility of the structures are obtained using only one or three coating layers.

In conclusion, we have designed and optimized the invisibility condition for all-dielectric multi-layer structures at a given frequency by employing a genetic algorithm. By using experimental data to describe materials, we have demonstrated it is possible to achieve nearly 80 fold suppression of total scattering cross section by using three or less dielectric layers. We have designed the optimized structures with minimal SCS for AlAs, TlBr and GaAs scatterers using alternating silicon and fused-silica coatings and proved that increasing the number of layers in the dielectric multi-layer shell, leads to saturation of the minimal scattering cross-section.

## Methods

**Figure 8:**
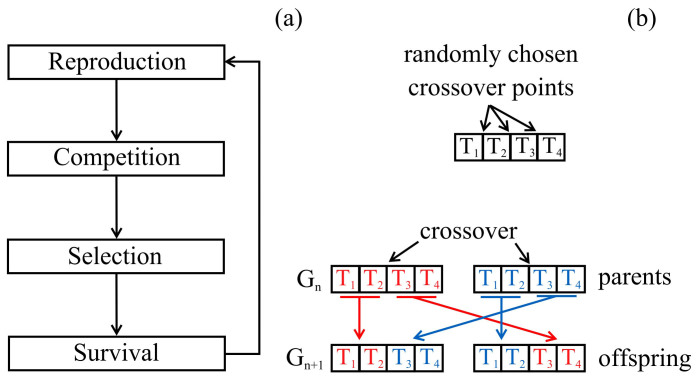
(a) General flow of a genetic algorithm, and (b) crossover process applied to a three-layer structure (two coating shells have four genes, two for radii and two for their materials). Chromosomes of parents are divided into two parts (head and tail) which are then swapped to generate offspring having their properties. The crossover point is chosen randomly. *T_m_* and *G_n_* indicate genes of chromosomes and *n*'th generation, respectively^36^.

Genetic algorithms (GAs)^29^,^30^,^31^,^32^ are based on random number generation and their aim is to start from a randomly generated ‘population’ of ‘individuals’ to reach an optimized population regarding to defined parameters and values to be optimized via regeneration. To characterize every individual (which is a multilayer structure here), we need a unique array of these parameters which is called ‘chromosome’. Every single one of these parameters is called a ‘gene’ (here radii and materials of every layer are genes). The first randomly generated population of structures is called the first ‘generation’. By choosing random pairs of individuals as ‘parents’ in each generation and generating their ‘offspring’, next generations are formed and this continues until we achieve some individuals which are sufficiently optimized. Figure 8(a) demonstrates the general flow of GAs.

New generations are born through some known procedures. In this work we use ‘crossover’ (as is demonstrated in Fig. 8(b)) as well as ‘mutation’ for regeneration. By applying crossover, different parts of the chromosomes are swapped to form new ones. Also mutation randomly changes a gene in a chromosome (for example in one layer of a multilayer structure, the radius can change randomly in an allowed range or a different material can be randomly chosen for it from a defined set of materials).

**Figure 9:**
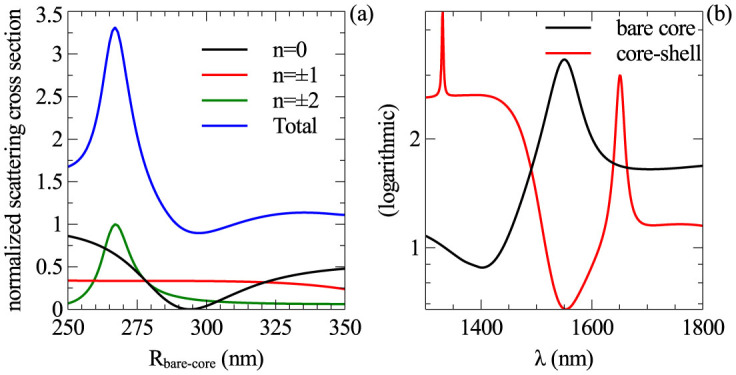
Cloaking a GaAs core with a Si shell under TM polarization (electric field parallel to the axis of the structure). (a) The radius of the bare core is chosen to have the maximum SCS (*R_core_* = 267 nm) to be cancelled (similar to Fig. 2), and (b) suppression of the total SCS by adding a Si shell similar to Fig. 3(c) (*R_total_* = 382 nm).

To employ our genetic algorithm to minimize the scattering cross section, we need to define a ‘fitness function’, as
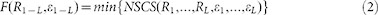
where *R_l_* represents the outer radius of the *l*-th layer. The GA searches the parameters space by calculating ‘fitness values’ and then removes and replaces the individuals with lower fitness values (the fitness value of an individual shows how close it is to the ideal properties the GA is looking for). In the discussed results, the fitness function is defined based on the NSCS in TE polarization by having magnetic field parallel to the axis of the nanowires. However, similar results can be obtained for TM polarization, as we demonstrate in Fig. 9 in ‘Additional information’ section.

In the discussion section, to calculate the stored energy in layer ‘*l*’ of a multi-layer nanowire, we use
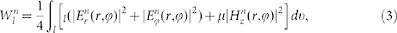
in which *n* is the mode number.

## Author Contributions

A.M. wrote the main manuscript and prepared the figures. A.E.M. and I.S. added more scientific detailed descriptions and completed the discussion section. A.E.M., I.S. and Y.K. reviewed and revised the manuscript. All authors discussed the results and contributed to the scientific interpretation.

## Figures and Tables

**Table 1:** Optimization of the invisibility of AlAs, TlBr and GaAs nanowires by multi-shell structures (silicon and fused-silica). The red numbers indicate repeated values which do not improve the optimization process as compared to previously found results. The green numbers represent the optimized SCS and size parameters. Adding more layers to the optimised shells does not reduce the SCS any further. The core radii *R*_1_ are chosen based on Fig. 2 to have the maximum SCS without dielectric shells. The superscript ‘§’ indicates the bare core while ‘^†^’ and ‘^‡^’ indicate the shells' sequence starting with silicon and fused-silica, respectively

Core	Config.	SCS	*R*_1_	*R*_2_	*R*_3_	*R*_4_	*R*_5_
	1^§^	2.575	303				
	2^†^	0.05	303	431			
	2^‡^	2.575	303	303			
	3^†^	0.05	303	431	431		
AlAs	3^‡^	0.036	303	309	432		
	4^†^	0.033	303	346	355	433	
	4^‡^	0.036	303	309	432	432	
	5^†^	0.033	303	346	355	433	433
	5^‡^	0.033	303	303	346	355	433
	1^§^	2.988	369				
	2^†^	0.359	369	496			
	2^‡^	2.988	369	369			
	3^†^	0.359	369	496	496		
TlBr	3^‡^	0.181	369	392	503		
	4^†^	0.170	369	402	426	504	
	4^‡^	0.181	369	392	503	503	
	5^†^	0.170	369	402	426	504	504
	5^‡^	0.170	369	369	402	426	504
	1^§^	2.353	261				
	2^†^	0.076	261	381			
GaAs	2^‡^	2.353	261	261			
	3^†^	0.076	261	381	381		
	3^‡^	0.076	261	261	381		
